# *Pleurotus ostreatus* for Environmental Remediation and Sustainable Bioprocesses: An Evidence-Mapped Review of Research Gaps and Opportunities

**DOI:** 10.3390/jof12010054

**Published:** 2026-01-12

**Authors:** Luz Miryam Lozada-Martinez, Juan David Reyes-Duque, Yadira Marin-Hamburger, Ivan David Lozada-Martinez

**Affiliations:** 1 Microbiology Program, Universidad Libre, Pereira 660004, Colombia; luzm-lozadam@unilibre.edu.co; 2 Facultad de Ciencias para la Salud, Universidad de Manizales, Manizales 170001, Colombia; jreyes@umanizales.edu.co; 3 Biomedical Scientometrics and Evidence-Based Research Unit, Department of Health Sciences, Universidad de la Costa, Barranquilla 08001, Colombia; ymarin3@cuc.edu.co; 4 Clínica Iberoamérica, Barranquilla 08001, Colombia

**Keywords:** *Pleurotus*, fungi, environment, sustainable development, knowledge bases, bibliometric

## Abstract

Fungi have emerged as versatile biotechnological platforms for addressing environmental challenges with potential co-benefits for human health. Among them, *Pleurotus ostreatus* stands out for its ligninolytic enzyme systems (notably laccases), capacity to valorize lignocellulosic residues, and ability to form functional mycelial materials. We conducted an evidence-mapped review, based on a bibliometric analysis of the Scopus corpus (2001–2025; 2085 records), to characterize research fronts and practical opportunities in environmental remediation and sustainable bioprocesses involving *P. ostreatus*. The mapped literature shows sustained growth and global engagement, with prominent themes in: (a) oxidative transformation of phenolic compounds, dyes and polycyclic aromatic hydrocarbons; (b) biodegradation/bioconversion of agro-industrial residues into value-added products; and (c) development of bio-based materials and processes aligned with the circular bioeconomy. We synthesize how these strands translate to real-world contexts, reducing contaminant loads, closing nutrient loops, and enabling low-cost processes that may indirectly reduce exposure-related risks. Key translational gaps persist: standardization of environmental endpoints, scale-up from laboratory to field, performance in complex matrices, life-cycle impacts and cost, ecotoxicological safety, and long-term monitoring. A practical agenda was proposed that prioritizes field-scale demonstrations with harmonized protocols, integration of life-cycle assessment and cost metrics, data sharing, and One Health frameworks linking environmental gains with plausible health co-benefits. In conclusion, *P. ostreatus* is a tractable platform organism for sustainable remediation and bio-manufacturing. This evidence map clarifies where the field is mature and where focused effort can accelerate the impact of future research.

## 1. Introduction

Fungi have long been recognized as key players in ecosystem functioning, with the capacity to transform organic matter, degrade complex polymers, and sustain nutrient cycles [1]. Over the last two decades, edible and medicinal mushrooms have moved beyond their traditional food role to become versatile platforms for biotechnology, environmental remediation, and sustainable materials [2]. Within this group, *Pleurotus ostreatus* (oyster mushroom) has attracted particular attention [3]. Its fast growth, low-cost cultivation, and ability to thrive on diverse lignocellulosic substrates make it a promising candidate for low-input bioprocesses [3]. Equally important, its enzymatic systems, especially laccases and other oxidoreductases, enable the breakdown of persistent pollutants such as dyes, phenolic compounds, polycyclic aromatic hydrocarbons, and plastics [4].

These attributes position *P. ostreatus* at the intersection of environmental science, food security, and human health [3,5]. On the one hand, it offers cost-effective tools for managing agro-industrial residues, mitigating environmental contamination, and developing bio-based alternatives that fit circular economy principles [6]. On the other hand, it contributes nutritional and bioactive compounds with potential co-benefits for population health [6].

Despite the growing interest in *P. ostreatus* as a platform organism, the field faces several persistent challenges that limit large-scale translation. These include the predominance of laboratory-based studies, fragmentation across disciplinary silos, lack of standardized environmental and health-related endpoints, limited integration of life-cycle and economic considerations, and uneven global participation in research and implementation [2]. At the same time, important opportunities are emerging, driven by advances in fungal biotechnology, circular bioeconomy frameworks, low-cost remediation needs in resource-limited settings, and increasing interest in One Health-oriented solutions [3].

Systematically identifying where these challenges and opportunities are concentrated within the existing literature is essential to guide future research, funding priorities, and policy-relevant applications [7,8,9]. Evidence mapping (a structured approach that systematically identifies, categorizes, and visually summarizes the extent, characteristics, and gaps of available evidence without quantitatively synthesizing outcomes) and bibliometric approaches (quantitative methods that analyze publication metadata, such as citations, keywords, and collaboration networks, to map research activity and thematic trends) provide a structured way to achieve this goal by quantitatively characterizing research fronts, gaps, and translational potential across scales and regions [7,8,9]. Such analyses can inform a forward-looking agenda for both scientific research and practical implementation [7,8].

Previously, the scientific growth and characteristics of this topic have never been quantified or analyzed. Therefore, the aim of this review is to map and critically appraise global research on *P. ostreatus*, synthesizing its contributions to environmental remediation and sustainable bioprocesses while identifying key knowledge gaps and opportunities for future application.

## 2. Materials and Methods

### 2.1. Study Design

We conducted an evidence-mapped review supported by bibliometric methods, designed to characterize the scientific landscape on *P. ostreatus* and its applications in environmental remediation and sustainable bioprocesses. This approach combines systematic retrieval of indexed literature with quantitative mapping of research activity, enabling the identification of thematic clusters, geographic patterns, and translational gaps relevant to environmental and human-health contexts [7,9,10].

### 2.2. Data Source

The Scopus database was selected given its broad coverage of peer-reviewed journals across environmental sciences, biotechnology, and health-related disciplines. Scopus also provides consistent metadata (authors, affiliations, citations, keywords, document types), which are essential for quantitative mapping and network analysis [11].

The decision to rely exclusively on the Scopus database was methodological and deliberate. Scopus offers broad and consistent coverage of peer-reviewed literature across environmental sciences, biotechnology, engineering, and applied mycology, which are central to the scope of this study. Importantly, Scopus provides standardized and complete bibliographic metadata (including citation counts, affiliations, keywords, and references), which are essential for robust bibliometric and evidence-mapping analyses [12]. In contrast, PubMed is primarily oriented toward biomedical indexing and lacks the comprehensive citation and affiliation data required for quantitative mapping while combining multiple databases may introduce duplication and inconsistencies that compromise network and trend analyses [13]. Therefore, the use of a single, well-established bibliometric database enhances internal consistency and reproducibility.

### 2.3. Search Strategy

A systematic search was performed using controlled descriptors and synonyms related to *P. ostreatus* (MeSH Unique ID: D020076). The final query was: TITLE (“*Pleurotus ostreatus*” OR “oyster mushrooms” OR “oyster mushroom”). No restrictions were applied by discipline, document type (other than inclusion criteria described below), or language, in order to capture the widest possible evidence base.

The title-restricted strategy was intentionally selected to maximize precision and ensure that *P. ostreatus* constituted the central focus of the retrieved documents. During piloting, expanding the query to abstract and keyword fields substantially increased the number of false positives, including records in which *P. ostreatus* was mentioned only incidentally (e.g., within mixed-species discussions, background statements, or broader mushroom biotechnology contexts). Because bibliometric mapping is highly sensitive to corpus noise, we prioritized a high-specificity dataset to improve the internal validity of collaboration and thematic network analyses.

### 2.4. Time Frame

The search covered publications available up to June 2025. Data collection and validation were performed between 18 June and 2 July 2025. This time frame allowed for consolidation of both historical contributions and the most recent advances.

### 2.5. Eligibility Criteria

Inclusion criteria were defined a priori as follows:

(i) Articles published in peer-reviewed journals;
(ii) Studies in which *P. ostreatus* was the central subject of investigation;
(iii) Availability of complete bibliographic metadata and at least an abstract in English or Spanish.

Exclusion criteria included:

(i) Studies referring to the genus *Pleurotus* without species-level specification of *P. ostreatus*;
(ii) Document types not suitable for bibliometric mapping (book chapters, conference proceedings, editorials, and errata);
(iii) Records lacking thematic relevance, defined as mentioning *P. ostreatus* only incidentally without substantive analytical focus.

These criteria were applied during a two-step screening process following data export from Scopus, ensuring consistency and reproducibility in the construction of the final evidence map.

### 2.6. Data Standardization

All records were exported in CSV format from Scopus, including full metadata fields. A two-step cleaning process was applied. First, duplicates and irrelevant records were removed manually by an independent reviewer. Second, terminology harmonization was conducted to consolidate synonyms, unify institutional names, and ensure conceptual consistency across keywords.

### 2.7. Data Analysis and Visualization

Bibliometric analyses were carried out using the Bibliometrix package in R (v4.5.1) [11] and VOSviewer (v1.6.20) [11]. Indicators included publication trends, citation metrics, institutional and country-level productivity, and co-authorship networks

Bibliometric mapping was conducted following a standardized, multi-step procedure to ensure transparency and reproducibility. First, cleaned and standardized Scopus records were imported into the Bibliometrix package (v4.5.1) for descriptive analyses and preliminary network construction. Annual publication trends, citation metrics, and productivity indicators were generated using default bibliometric functions.

This includes the description of baseline structural characteristics of the scientific corpus. These characteristics encompass the volume and temporal distribution of publications, the diversity of publication sources, citation patterns, and authorship and collaboration structures. Such variables are routinely reported in scientometrics studies to contextualize the size, maturity, interdisciplinarity, and collaborative dynamics of a research field prior to interpretative mapping. Rather than assessing biological performance or technological effectiveness, these descriptors provide a foundational understanding of how knowledge on *P. ostreatus* is produced, organized, and disseminated, thereby informing the interpretation of subsequent thematic and relational analyses.

Countries were grouped into broad geographical regions (Americas, Europe, Western Pacific, Eastern Mediterranean, Southeast Asia, and Africa) [14] and categorized by income level (low, lower-middle, upper-middle, and high) [14]. Importantly, the income classification strictly followed the official World Bank classification system [14], ensuring methodological consistency and international comparability.

For network-based visualizations, VOSviewer (v1.6.20) was used to construct co-authorship and keyword co-occurrence maps. Keyword co-occurrence analysis was performed using authors’ keywords and Keywords Plus, applying a minimum occurrence threshold of five to reduce noise and improve interpretability. Networks were normalized using the association strength method, and clusters were identified using the VOS clustering algorithm with default resolution parameters, which are commonly applied in bibliometric mapping studies.

Temporal trends in thematic evolution were assessed by overlay visualization, allowing for identification of emerging and declining topics over time. All parameter settings were selected to balance analytical robustness with visual clarity and are consistent with established practices in scientometrics research.

## 3. Results

### 3.1. Publication Trends and Global Productivity

**Figure 1:**
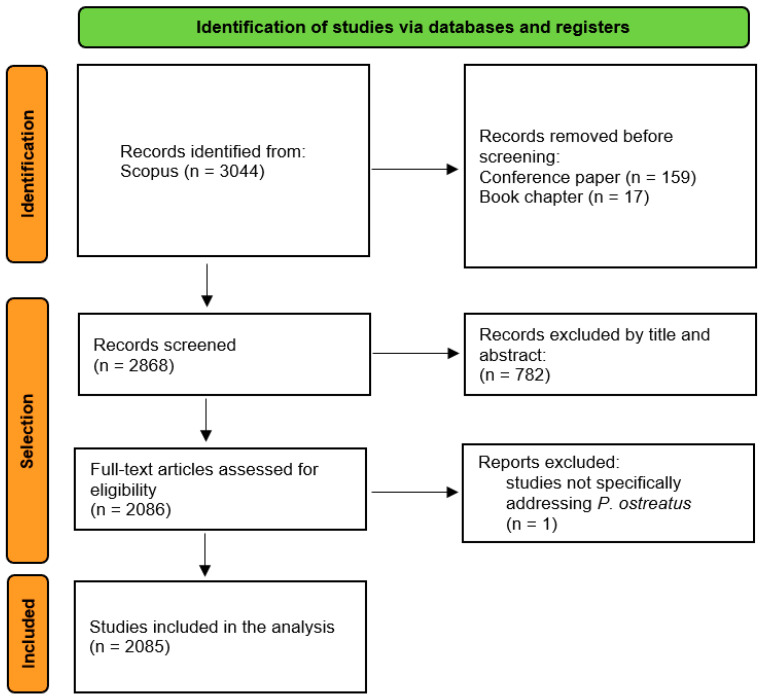
Flowchart of study selection.

**Figure 2:**
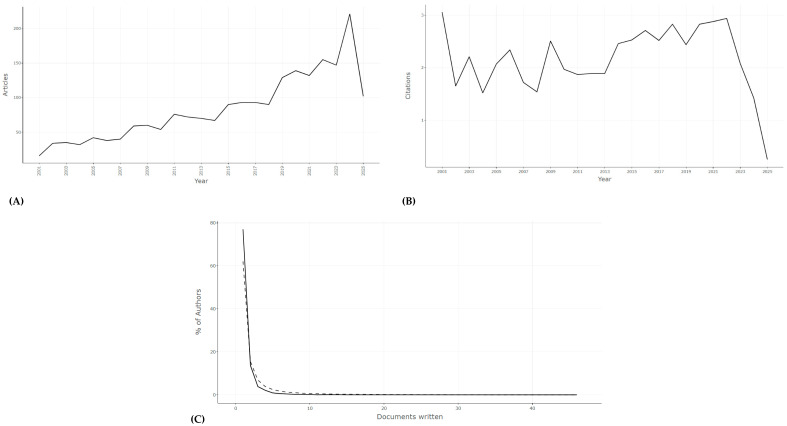
Annual growth of global research on *P. ostreatus*. (**A**) Number of publications per year. (**B**) Mean total citations per article per year. (**C**) Author productivity distribution according to Lotka’s law. The data demonstrate sustained growth in publications and highlight a small set of influential studies driving the field.

Between 2001 and 2025, we identified 2085 peer-reviewed publications on *P. ostreatus* (Figure 1), with an average annual growth rate of over 8% (Table 1). The increasing trajectory, particularly after 2010 (Figure 2A–C), reflects the consolidation of this species as a model organism for biotechnological and environmental applications. The majority of contributions came from China, India, and the United States (Table 2), complemented by significant outputs from Latin America and Europe. This global distribution highlights a dual dynamic: highly resourced research hubs in Asia driving scale and productivity, and emerging regions contributing context-specific applications, often linked to low-cost remediation or waste valorization.

From an environmental standpoint, the expansion of *P. ostreatus* research mirrors global interest in circular bioeconomy strategies. The steady rise in publications underscores the recognition of fungi not only as biological curiosities but as platforms for scalable solutions to pollution, waste management, and sustainable production systems.

### 3.2. Regional Patterns and Socioeconomic Context

Regional analysis revealed that Europe and Asia produced the largest volume of outputs, with Europe showing the highest h-index (n = 69), reflecting both productivity and citation impact. Latin America and the Caribbean contributed nearly 15% of the global corpus (Table 3), often focusing on agro-industrial waste valorization and soil bioremediation, which directly align with regional sustainability challenges. Importantly, income-based stratification, applied using the official World Bank classification, demonstrated that upper-middle and high-income economies dominate the field (Table 4). However, the active participation of lower- and lower-middle-income economies, particularly in Asia and Africa, illustrates how *P. ostreatus* serves as a cost-effective platform for research and innovation in resource-limited settings.

This socioeconomic pattern is critical: while wealthier economies drive innovation in enzyme purification, genomics, and advanced materials, lower-income contexts showcase practical implementations such as the bioremediation of hydrocarbons, dye decolorization, and low-cost mushroom farming using local residues. Together, these findings underscore the adaptability of *P. ostreatus* to different environmental and socioeconomic realities.

### 3.3. Institutional Leadership

**Figure 3:**
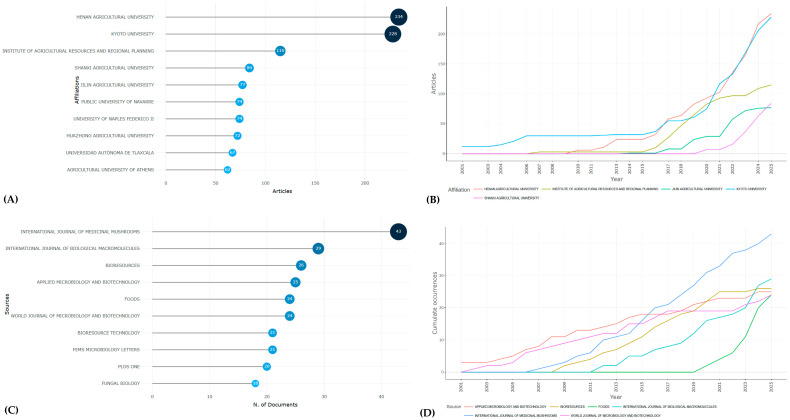
Institutional and journal contributions to *P. ostreatus* research. (**A**) Most productive affiliations. (**B**) Institutional output trends over time. (**C**) Journals with the largest number of publications. (**D**) Journal production trends over time. The results reveal that East Asian universities dominate global output, while specialized journals in biotechnology and fungal biology provide key publication platforms.

Collaboration mapping indicated a strong concentration of research activity in East Asia, particularly within Chinese and Japanese institutions, with Henan Agricultural University and Kyoto University among the most productive (Figure 3A,B).

Institutional leadership also influences thematic focus. Asian institutions tend to prioritize enzyme optimization and industrial applications, while Latin American centers emphasize agro-industrial waste management and soil remediation. This thematic divergence reflects how regional priorities shape scientific agendas, but it also reveals opportunities for more integrated, global strategies.

### 3.4. Thematic Hotspots and Knowledge Clusters

The *International Journal of Medicinal Mushrooms* and the *International Journal of Biological Macromolecules* emerged as the most frequent sources for studies on *P. ostreatus*. Figure 3C shows that these journals, along with others such as *Bioresources*, *Applied Microbiology and Biotechnology*, and *Fungal Biology*, have provided consistent platforms for dissemination. Figure 3D illustrates a steady rise in journal productivity over the past two decades, reflecting the diversification of research areas, from fungal physiology and biochemistry to applied biotechnology and environmental sciences. This trend highlights the multidisciplinary nature of *P. ostreatus* research and its integration into both specialized and broad-scope journals.

**Figure 4:**
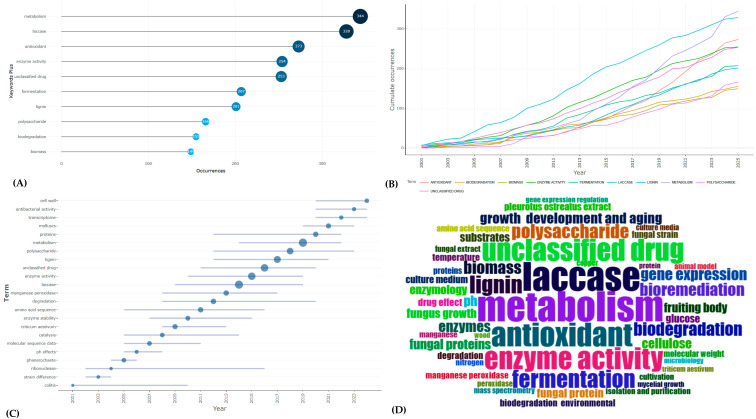
Thematic evolution of research on *P. ostreatus*. (**A**) Most frequent author keywords. (**B**) Temporal evolution of keywords. (**C**) Trending research topics by year. (**D**) Word cloud of thematic focus. Thematic clusters emphasize enzymatic activity, biodegradation, and antioxidant potential, with a growing diversification into molecular and material science applications.

Keyword co-occurrence analysis revealed three dominant thematic clusters: (a) enzymatic degradation and oxidative processes, with a strong focus on laccase activity; (b) bioconversion of lignocellulosic substrates into biofuels, biopolymers, or functional foods; and (c) environmental remediation of pollutants such as phenolics, dyes, and polycyclic aromatic hydrocarbons (Figure 4A,B). Recent trends show an expansion towards emerging topics such as microplastic degradation, transcriptomics, and mycelium-based materials (Figure 4C,D).

From an applied perspective, these clusters align closely with global environmental priorities: reducing industrial pollution, valorizing waste streams, and developing sustainable alternatives to fossil-based materials. However, gaps remain in translating laboratory findings into large-scale field demonstrations, as well as in evaluating long-term environmental safety and health co-benefits.

### 3.5. Collaboration Networks

**Figure 5:**
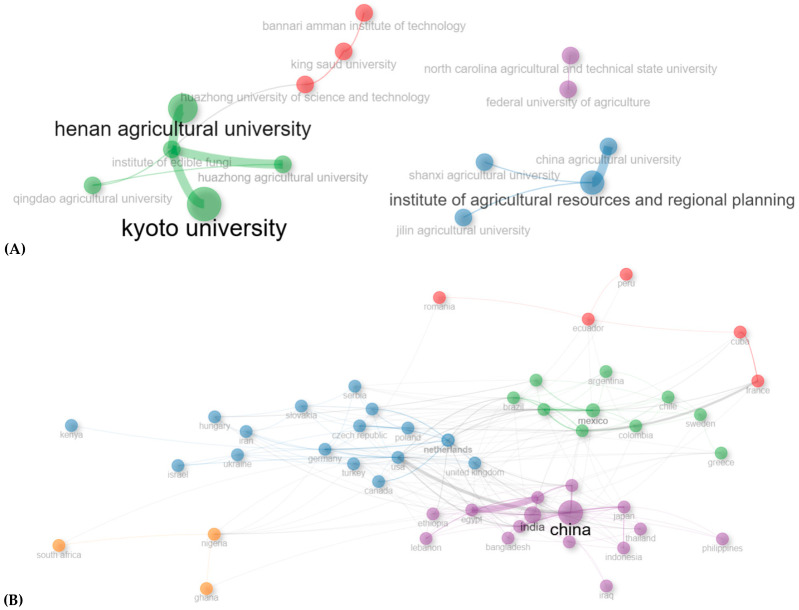
Collaboration networks in *P. ostreatus* research. (**A**) Institutional collaboration patterns. (**B**) International co-authorship by country. Strong clusters are evident in East Asia, particularly among Chinese and Japanese institutions, while Latin America forms a cohesive collaboration network led by Mexico. Emerging but weaker links are observed in Africa.

International collaboration was present but uneven, with lower representation from Africa and underdeveloped regions. Strengthening South–South and North–South collaborations could accelerate technology transfer and contextual adaptation, particularly for waste-to-resource applications in low- and middle-income countries (Figure 5A,B).

## 4. Discussion

Although global research on *P. ostreatus* has expanded rapidly and diversified thematically [6,15], several challenges remain before its potential for environmental remediation and sustainable bioprocesses can be fully realized. These challenges reflect both scientific gaps and structural barriers in the way research is conducted, reported, and translated into practice [16,17,18]).

### 4.1. Limited Translation from Laboratory to Field

Most studies have been carried out under controlled laboratory conditions, often using simplified substrates or pollutant concentrations that do not reflect real environmental complexity [19]. While these experiments provide valuable mechanistic insights, they rarely capture the variability in soils, effluents, or agro-industrial residues in situ. Field-scale demonstrations remain scarce, particularly in low- and middle-income countries where the technology could be most impactful [19]. Without such evidence, it is difficult to evaluate the robustness and reproducibility of *P. ostreatus* applications in heterogeneous and dynamic environments.

### 4.2. Fragmentation of Thematic Approaches

The bibliometric mapping revealed distinct clusters, enzymatic degradation, waste valorization, bio-based materials, that are rarely integrated in a single research agenda. For instance, work on laccase optimization often proceeds independently of studies on agro-industrial waste remediation, despite obvious synergies [20]. This thematic fragmentation limits the ability to design multi-functional bioprocesses that combine pollution reduction, waste management, and sustainable production [20].

### 4.3. Interrelationships, Complementarities, and Constraints Across Thematic Clusters

The three dominant thematic clusters identified in this evidence map, enzymatic degradation, waste valorization, and bio-based materials, should not be interpreted as isolated research streams, but as interrelated components of *P. ostreatus* biotechnology. Historically, early research focused on ligninolytic enzyme systems, particularly laccases, which established the biochemical basis for pollutant degradation and oxidative transformation [21]. These enzymatic capabilities subsequently enabled applications in agro-industrial waste valorization, where substrate composition, nutrient availability, and fungal physiology directly influence both remediation efficiency and biomass production [21].

More recently, advances in mycelium-based materials and biomaterials research have drawn upon insights from both enzymatic optimization and substrate engineering, illustrating a convergence rather than a divergence of these clusters [22]. However, this integration also exposes key trade-offs specific to *P. ostreatus*. Conditions that maximize enzymatic activity do not always align with those required for stable mycelial growth or material formation, and substrate heterogeneity can differentially affect degradation performance versus biomass quality [22].

These interdependencies highlight organism-specific constraints that shape translational potential. The need to balance enzymatic efficiency, substrate adaptability, and structural integrity of mycelial products represents a central biotechnological challenge for *P. ostreatus*. The limited number of studies explicitly addressing these trade-offs across clusters underscores a critical gap in the literature, reinforcing the value of an evidence-mapped approach to identify where integration remains underexplored.

### 4.4. Gaps in Standardized Environmental and Health Endpoints

Studies frequently report pollutant removal or enzyme activity using non-standardized protocols, which complicates cross-comparison and meta-analysis [23]. Moreover, few investigations link environmental endpoints (e.g., contaminant degradation, soil quality) to human-health outcomes (e.g., reduced exposure pathways, nutritional improvements from valorized products) [23]. The absence of harmonized benchmarks restricts the capacity to position *P. ostreatus* interventions within broader One Health frameworks [23].

### 4.5. Socioeconomic Inequalities and Lack of Collaboration

Our results show that high- and upper-middle-income economies dominate research output and citation impact, while contributions from Africa and other resource-limited settings remain marginal [24]. This imbalance risks reinforcing technological dependency and missing context-specific innovations [24]. International collaboration networks, although growing, are still heavily centered in East Asia, leaving limited opportunities for knowledge transfer and capacity building in emerging regions.

### 4.6. Insufficient Integration of Life-Cycle and Cost Analyses

While environmental benefits are often demonstrated at a technical level, few studies evaluate the economic feasibility, energy balance, or long-term sustainability of *P. ostreatus*-based interventions. Life-cycle assessment and cost-effectiveness analysis are largely absent from the literature, creating uncertainty about scalability and competitiveness compared with conventional technologies [25]. Without such assessments, policymakers and industries may be reluctant to adopt fungal bioprocesses at scale [25].

### 4.7. Emerging but Underexplored Areas

Recent keywords and citation bursts point to novel applications such as microplastic degradation, mycelium-based materials, and bioactive metabolite discovery [26]. However, these remain at exploratory stages, with few validated protocols or comparative studies. Establishing the ecological safety, durability, and performance of these applications requires systematic investigation before they can be integrated into mainstream environmental practice [26,27].

### 4.8. Data Accessibility and Methodological Transparency

Although bibliometric growth is evident, data sharing and open protocols remain limited. Raw datasets on degradation rates, enzyme kinetics, and long-term field trials are seldom made publicly available. This restricts reproducibility and slows the accumulation of robust, comparable evidence [7,8]. Greater transparency is needed to accelerate innovation and avoid duplication of effort across regions and disciplines [7,8].

### 4.9. Future Directions

The future directions outlined below are not intended as generic recommendations, but as a prioritized and organism-specific roadmap derived from the biological, technological, and translational characteristics of *P. ostreatus* identified through the evidence mapping. In particular, they reflect recurrent bottlenecks observed in the literature, including the gap between laboratory enzymatic performance and field robustness, the context-dependence of substrate-fungus interactions, and the limited integration of standardized endpoints and scalability considerations. The proposed directions are therefore ordered to address these constraints sequentially, from biological validation under real conditions to system-level integration and policy relevance.

The growing body of research on *P. ostreatus* provides a strong foundation for its role in environmental remediation and sustainable bioprocesses. However, to move beyond proof-of-concept studies and ensure measurable environmental and health benefits, future efforts should prioritize the following directions:

### 4.10. Field Validation and Scale-Up

Large-scale pilot studies in real environments are urgently needed to validate laboratory findings under complex ecological conditions [28]. This includes contaminated soils, industrial effluents, and mixed agro-industrial residues. Standardized protocols for monitoring pollutant removal, soil quality, and long-term stability should be adopted to enable comparability across sites and contexts [28].

### 4.11. Harmonization of Endpoints

Developing consensus on performance indicators, both environmental (e.g., degradation efficiency, carbon footprint, nutrient cycling) and health-related (e.g., reduction in exposure to pollutants, nutritional safety of valorized products), will be essential [29]. Establishing harmonized benchmarks will allow for integration of *P. ostreatus* into international frameworks such as One Health and circular economy policies.

### 4.12. Integration of Life-Cycle and Economic Assessments

Future studies should systematically incorporate life-cycle assessment and cost-effectiveness analyses [25]. This will provide realistic estimates of environmental gains, energy balances, and economic competitiveness compared with conventional remediation and waste management technologies [25]. Such evidence is critical for convincing policymakers and industries to adopt fungal-based solutions [25].

### 4.13. Strengthening Global Collaboration

Expanding research networks beyond established hubs in East Asia and Europe is essential to ensure global relevance [30]. North–South and South–South collaborations should be prioritized, enabling capacity building in low- and middle-income countries [31]. This will foster innovations tailored to local environmental challenges [31], such as hydrocarbon-contaminated soils in Africa or agro-industrial residues in Latin America.

### 4.14. Advancing Underexplored Applications

Emerging areas such as microplastic degradation, mycelium-based materials, and novel bioactive metabolites merit systematic exploration [26,28]. These fields hold promise for addressing global environmental and health challenges, but require robust validation of safety, durability, and performance under realistic conditions [26,28]. Interdisciplinary projects bridging environmental chemistry, toxicology, and material sciences will be key 26,28].

While this review does not aim to systematically quantify performance metrics across heterogeneous experimental designs, several published studies illustrate the successful practical application of *P. ostreatus* under diverse environmental conditions. For example, field and pilot-scale studies have reported effective decolorization of textile dyes and phenolic compounds in industrial effluents, with removal efficiencies frequently exceeding 70–90% under optimized aeration, pH, and substrate conditions [32]. Similarly, soil-based applications in hydrocarbon-contaminated environments have demonstrated substantial reductions in total petroleum hydrocarbons when *P. ostreatus* is cultivated on locally available lignocellulosic residues, highlighting its adaptability to complex matrices [33].

In agro-industrial contexts, *P. ostreatus* has been successfully deployed for the bioconversion of residues such as coffee pulp, brewer’s spent grain, and agricultural straw, yielding both edible biomass and value-added byproducts while maintaining stable performance across a range of temperatures (approximately 20–30 °C) and moisture conditions [34]. These examples underscore that, although performance varies according to substrate composition, pollutant load, and environmental parameters, *P. ostreatus* consistently demonstrates functional robustness in non-laboratory settings.

Importantly, these practical cases also reveal a major knowledge gap: performance data are often reported using non-harmonized endpoints and context-specific metrics, which limits cross-study comparison and scalability assessment. This reinforces the need for standardized reporting frameworks rather than additional isolated case studies.

### 4.15. Context-Sensitive Policy Implications

The evidence map highlights that *P. ostreatus* research and applications are shaped by distinct regional and socioeconomic contexts, which implies that policy priorities should be context-sensitive rather than uniform [35]. In high-income economies, where research capacity and industrial infrastructure are well established, policy efforts may focus on supporting scale-up, standardization, and regulatory pathways for advanced biotechnological applications, including enzyme production, mycelium-based materials, and integration into circular bioeconomy strategies [35].

In upper- and lower-middle-income economies, where much of the research activity is concentrated on agro-industrial waste valorization and low-cost remediation, policies could prioritize pilot-scale demonstrations, public-private partnerships, and incentives for locally adapted bioprocesses that link environmental remediation with value-added production [36]. In low-income and resource-limited settings, the mapped literature suggests opportunities for policies that support capacity building, technology transfer, and community-based applications of *P. ostreatus*, particularly for soil remediation and residue management using locally available substrates [36].

Across all regions, a common policy-relevant gap identified by this review is the lack of harmonized performance, safety, and sustainability metrics. Policies that encourage standardized reporting, long-term monitoring, and integration of life-cycle and economic assessments would facilitate comparability and evidence-based decision-making, thereby accelerating responsible adoption of fungal-based solutions.

### 4.16. Promoting Data Sharing and Open Science

To accelerate progress, future work should adopt open science practices, including public repositories for degradation data, enzyme kinetics, and field trial results [37]. Shared datasets and transparent methodologies will reduce duplication, enhance reproducibility, and enable meta-analyses [37] that clarify where *P. ostreatus* interventions are most effective.

**Figure 6:**
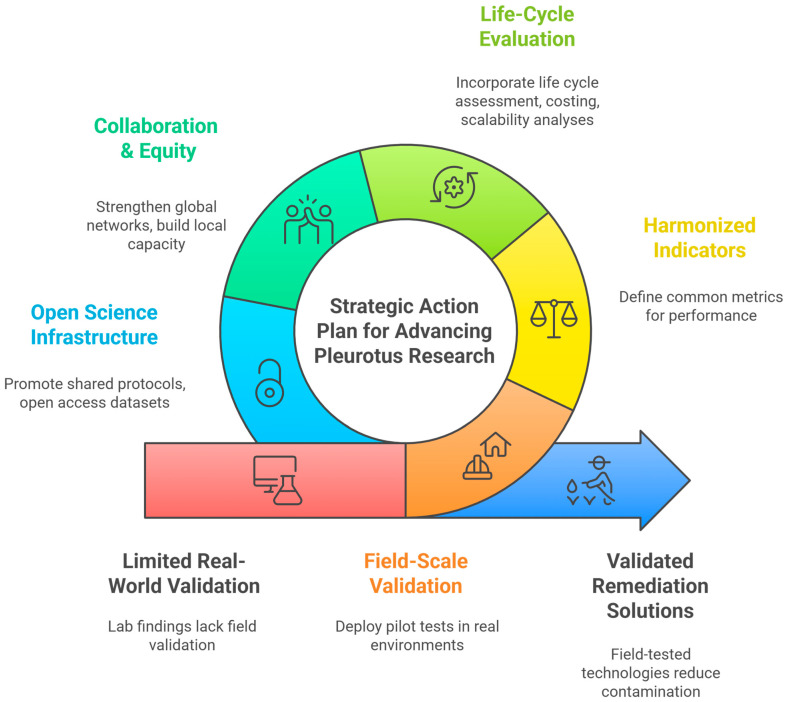
Strategic conceptual-practical action plan for advancing *P. ostreatus* research toward environmental remediation, sustainable bioprocesses, and one health integration. This figure illustrates an integrated conceptual and practical roadmap for accelerating the translational impact of *P. ostreatus*. Three core pillars: environmental remediation, sustainable bioprocesses, and potential One Health co-benefits, form the foundation of the framework. Surrounding these pillars are the key translational gaps identified in the evidence-mapped review, including the laboratory-to-field gap, lack of standardized environmental and health endpoints, insufficient life-cycle and cost analyses, limited global collaboration, and restricted data sharing. The outer action layer details the strategic priorities required to overcome these barriers: field-scale validation, harmonized indicators, integration of life-cycle and economic assessments, strengthened international and equitable collaboration networks, and adoption of open-science practices. Together, this framework provides a structured agenda to guide future research, policy development, and biotechnological innovation involving *P. ostreatus*. Source: authors.

In summary, future research should focus on bridging the gap between laboratory innovation and field implementation, supported by harmonized methodologies, robust economic and life-cycle evidence, and stronger global collaboration (Figure 6). By following this roadmap, *P. ostreatus* can evolve from a promising model organism into a validated platform for sustainable environmental remediation and bio-based innovation, with tangible benefits for both ecosystems and human health.

From a One Health perspective, future studies would benefit from explicitly linking environmental remediation outcomes to measurable human health-relevant indicators. Examples of such indicators include reductions in environmental biomarkers of exposure (e.g., concentrations of polycyclic aromatic hydrocarbons or phenolic compounds in soil, water, or food matrices), changes in dietary exposure associated with the valorization of remediated substrates, and nutritional or safety assessments of *P. ostreatus* biomass produced under remediation scenarios. In occupational or community-based implementations, intermediate indicators such as reduced contact with contaminated substrates or improved local waste management practices could also be considered.

## 5. Conclusions

This evidence-mapped review confirms that *P. ostreatus* has emerged as a versatile platform organism with significant potential for environmental remediation and sustainable bioprocesses. Global research activity has expanded steadily, particularly in Asia and Latin America, with strong thematic foci on enzymatic degradation, agro-industrial waste valorization, and bio-based material development. Yet, despite this momentum, critical gaps persist: most studies remain confined to laboratory settings, standardized endpoints are lacking, and socioeconomic inequalities limit equitable adoption across regions.

Looking ahead, the field must prioritize field-scale demonstrations, harmonization of environmental and health-related benchmarks, integration of life-cycle and cost analyses, and broader international collaboration. By addressing these gaps, *P. ostreatus* can transition from a promising research model to a validated biotechnological solution that contributes directly to pollution reduction, resource recovery, and healthier environments. Ultimately, the challenge is not only to expand scientific knowledge but to ensure that it translates into tangible, scalable benefits for ecosystems and human well-being.

## Figures and Tables

**Table 1:** Basic characteristics of global research on *P. ostreatus*.

Indicator	Value	Percentage
Time period	2001–2025	-
Sources	861	-
Documents	2085	-
Annual growth (%)	8.02	-
Average document age (years)	8.31	-
Average citations per document	20.61	-
Total references	74,815	-
Keywords Plus (ID)	10,387	-
Authors’ keywords (DE)	4668	-
Total authors	6965	-
Single-authored documents	45	-
Authors of single-authored papers	38	-
Co-authors per document	5.14	-
International co-authorship (%)	17.5	-
**Document types**		
Articles	2042	97.94
Reviews	32	1.54
Notes	5	0.24
Short surveys	3	0.15
Data paper	2	0.09
Letter	1	0.04

**Table 2:** Top countries by number of publications and h-index.

Country	Documents	h-Index
China	386	48
India	267	42
Mexico	132	33
Japan	121	34
Brazil	116	31
United States	105	32
South Korea	109	28
Italy	98	35
Malaysia	104	27
Indonesia	57	13

**Table 3:** Regions by World Bank group with the highest number of frequency of co-authorships and h-index.

Region	Documents	h-Index
Central Europe and Asia	663	69
East Asia and Pacific	490	47
Southeast Asia	367	47
Latin America and the Caribbean	314	44
Middle East and North Africa	191	39
North America	117	34
Sub-Saharan Africa	164	25

**Table 4:** Frequency of co-authorships and h-index by World Bank income group.

Income Group (World Bank)	Documents	h-Index
Low income	31	14
Lower-middle income	589	53
Upper-middle income	1064	70
High income	985	79

## Data Availability

No new data were created or analyzed in this study. Data sharing is not applicable to this article.

## References

[1] Corbu V.M., Gheorghe-Barbu I., Dumbravă A.Ș., Vrâncianu C.O., Șesan T.E. (2023). Current insights in fungal importance—A comprehensive review. Microorganisms.

[2] Lange L. (2014). The importance of fungi and mycology for addressing major global challenges. IMA Fungus.

[3] Nakazawa T., Kawauchi M., Otsuka Y., Han J., Koshi D., Schiphof K., Ramírez L., Pisabarro A.G., Honda Y. (2024). *Pleurotus ostreatus* as a model mushroom in genetics, cell biology, and material sciences. Appl. Microbiol. Biotechnol..

[4] Shin H.J., Ro H.S., Kawauchi M., Honda Y. (2025). Review on mushroom mycelium-based products and their production process: From upstream to downstream. Bioresour. Bioprocess..

[5] do Nascimento Deschamps J.L., Schulz J.G., Riani J.C., Bonatti-Chaves M., Bonatti M., Sieber S., Lana M., Wisbeck E. (2024). Sustainable production of *Pleurotus sajor-caju* mushrooms and biocomposites using brewer’s spent and agro-industrial residues. Sci. Rep..

[6] Silva M., Ramos A.C., Lidon F.J., Reboredo F.H., Gonçalves E.M. (2024). Pre- and postharvest strategies for *Pleurotus ostreatus* mushroom in a circular economy approach. Foods.

[7] Lozada-Martínez I.D., Hernández-Páez D., Zárate Y.E.J., Delgado P. (2025). Scientometrics and meta-research in medical research: Approaches required to ensure scientific rigor in an era of massive low-quality research. Rev. Assoc. Med. Bras..

[8] Lozada-Martínez I.D., Hernández-Paz D.A., Fiorillo-Moreno O., Picón-Jaimes Y.A., Bermúdez V. (2025). Meta-research in biomedical investigation: Gaps and opportunities based on meta-research publications and global indicators in health, science, and human development. Publications.

[9] Lozada-Martínez I.D., Neira-Rodado D., Martinez-Guevara D., Cruz-Soto H.S., Sanchez-Echeverry M.P., Liscano Y. (2025). Why is it important to implement meta-research in universities and institutes with medical research activities?. Front. Res. Metr. Anal..

[10] Abramo G., D’Angelo C.A., Reale E. (2019). Peer review versus bibliometrics: Which method better predicts the scholarly impact of publications?. Scientometrics.

[11] Arruda H., Silva E.R., Lessa M., Proença D., Bartholo R. (2022). VOSviewer and Bibliometrix. J. Med. Libr. Assoc..

[12] Mongeon P., Paul-Hus A. (2016). The journal coverage of Web of Science and Scopus: A comparative analysis. Scientometrics.

[13] Gavel Y., Iselid L. (2008). Web of Science and Scopus: A journal title overlap study. Online Inf. Rev..

[14] The World Bank (2025). World Bank Country and Lending Groups. Data Help Desk. https://datahelpdesk.worldbank.org/knowledgebase/articles/906519-world-bank-country-and-lending-groups.

[15] Carrasco-Cabrera C.P., Bell T.L., Kertesz M.A. (2019). Caffeine metabolism during cultivation of oyster mushroom (*Pleurotus ostreatus*) with spent coffee grounds. Appl. Microbiol. Biotechnol..

[16] Elhamouly N.A., Hewedy O.A., Zaitoon A., Miraples A., Elshorbagy O.T., Hussien S., El-Tahan A., Peng D. (2022). The hidden power of secondary metabolites in plant-fungi interactions and sustainable phytoremediation. Front. Plant Sci..

[17] Kong H.H., Segre J.A. (2020). Cultivating fungal research. Science.

[18] Iliev I.D., Brown G.D., Bacher P., Gaffen S.L., Heitman J., Klein B.S., Lionakis M.S. (2024). Focus on fungi. Cell.

[19] Ioachimescu O.C., Shaker R. (2025). Translational science and related disciplines. J. Investig. Med..

[20] Balietti S., Mäs M., Helbing D. (2015). On disciplinary fragmentation and scientific progress. PLoS ONE.

[21] Parmar M., Patel S.A.H., Phutela U.G., Dhawan M. (2024). Comparative Analysis of Ligninolytic Potential among *Pleurotus ostreatus* and *Fusarium* sp. with a Special Focus on Versatile Peroxidase. Appl. Microbiol..

[22] Ruggeri M., Miele D., Contardi M., Vigani B., Boselli C., Icaro Cornaglia A., Rossi S., Suarato G., Athanassiou A., Sandri G. (2023). Mycelium-based biomaterials as smart devices for skin wound healing. Front. Bioeng. Biotechnol..

[23] Smith N.W., McDowell R.W., Smith C., Foster M., Eason C., Stephens M., McNabb W.C. (2025). Gaps in environmental and social evidence base are holding back strategic action on our national food system. J. R. Soc. N. Z..

[24] van Alderwick H., Hutchings A., Mays N. (2024). Cross-sector collaboration to reduce health inequalities: A qualitative study of local collaboration between health care, social services, and other sectors under health system reforms in England. BMC Public Health.

[25] Dong Y., Zhao Y., Wang H., Liu P., He Y., Lin G. (2022). Integration of life cycle assessment and life cycle costing for the eco-design of rubber products. Sci. Rep..

[26] Linder N., Giusti M., Samuelsson K., Barthel S. (2022). Pro-environmental habits: An underexplored research agenda in sustainability science. Ambio.

[27] Pérez-Fontalvo N.M., De Arco-Aragón M.A., Jimenez-García J.D.C., Lozada-Martinez I.D. (2021). Molecular and computational research in low- and middle-income countries: Development is close at hand. J. Taibah Univ. Med. Sci..

[28] Pearson N., Naylor P.J., Ashe M.C., Fernandez M., Yoong S.L., Wolfenden L. (2020). Guidance for conducting feasibility and pilot studies for implementation trials. Pilot. Feasibility Stud..

[29] Cooke S.J., Cook C.N., Nguyen V.M., Walsh J.C., Young N., Cvitanovic C., Grainger M.J., Randall N.P., Muir M., Kadykalo A.N. (2023). Environmental evidence in action: On the science and practice of evidence synthesis and evidence-based decision-making. Environ. Evid..

[30] Miranda-Pacheco J.A., De Santis-Tamara S.A., Parra-Pinzón S.L., González-Monterroza J.J., Lozada-Martínez I.D. (2021). Medical interest groups and work policies as emerging determinants of a successful career: A student perspective—Correspondence. Int. J. Surg..

[31] Bertuol-Garcia D., Morsello C., El-Hani C.N., Pardini R. (2018). A conceptual framework for understanding the perspectives on the causes of the science-practice gap in ecology and conservation. Biol. Rev. Camb. Philos. Soc..

[32] Rodríguez Pérez S., García Oduardo N., Bermúdez Savón R.C., Fernández Boizán M., Augur C. (2008). Decolourisation of mushroom farm wastewater by *Pleurotus ostreatus*. Biodegradation.

[33] Pozdniakova N.N., Nikitina V.E., Turkovskaia O.V. (2008). Bioremediation of oil-polluted soil with an association including the fungus *Pleurotus ostreatus* and soil microflora. Prikl. Biokhim Mikrobiol..

[34] Rivera-Hoyos C.M., Morales-Álvarez E.D., Abelló-Esparza J., Buitrago-Pérez D.F., Martínez-Aldana N., Salcedo-Reyes J.C., Poutou-Piñales R.A., Pedroza-Rodríguez A.M. (2018). Detoxification of pulping black liquor with *Pleurotus ostreatus* or recombinant Pichia pastoris followed by CuO/TiO_2_/visible photocatalysis. Sci. Rep..

[35] AlHamawi R., Saad R.K., Abdul Rahim H.F., Mir Islam Saeed K., Husseini A., Khader Y., Al Nsour M. (2023). Supporting Public Health Research Capacity, Quality, and Productivity in a Diverse Region. Interact. J. Med. Res..

[36] Iizumi T., Sakai T., Masaki Y., Oyoshi K., Takimoto T., Shiogama H., Imada Y., Makowski D. (2025). Assessing the capacity of agricultural research and development to increase the stability of global crop yields under climate change. PNAS Nexus.

[37] Staunton C., Barragán C.A., Canali S., Ho C., Leonelli S., Mayernik M., Prainsack B., Wonkham A. (2021). Open science, data sharing and solidarity: Who benefits?. Hist. Philos. Life Sci..

